# Pediatric Obesity: An Economic Perspective

**DOI:** 10.3389/fpubh.2020.619647

**Published:** 2021-01-08

**Authors:** Nathan Montoya Albrecht, Bashyam S. Iyengar

**Affiliations:** ^1^Department of Pediatrics, University of Florida College of Medicine, Jacksonville, FL, United States; ^2^Department of Family Medicine, Saint Vincent's Health Center, Jacksonville, FL, United States

**Keywords:** pediatric obesity, health economics, sugar tax, fast food marketing, advertising bans

## Introduction

Pediatric obesity is increasingly common in the United States, with over thirty percent of American children considered obese (BMI over the 95th percentile for age); and over forty percent of American adolescents classified as overweight (BMI between the 85th and 95th percentile for age) (1). The most serious projections estimate that over eighty-five percent of American adults will be overweight or obese by 2030 (2). Childhood obesity is now considered a growing epidemic requiring intervention and preventative measures, similar to tobacco use (3).

Childhood obesity affects every single organ system (3). Obesity is associated with concomitant or increased risk of nearly every chronic disease and condition, from diabetes to dyslipidemia, to cancers and poor mental health (4). The longer a person is obese the more compounded the costs from obesity's associated comorbidities, hypertension, hyperlipidemia, type 2 diabetes mellitus, obstructive sleep apnea, hepatic steatosis, orthopedic conditions, certain cancer, depression, and social isolation (5).

In the past, the public health community demonstrated that public smoking bans, advertising bans and increased taxation on cigarettes worked to decrease the general use of tobacco in the United States. The same policies could work when applied to the fast-food industry. These interventions act as economic disincentives and reduce the triggers that influence consumption without outright bans on fast food products.

## Behavioral and Classical Economics

John et al. (6) make the distinction between traditional economic theory and the field of behavioral economics in that behavioral economics attempts to reconcile psychological “errors” such as short-term time preferences, loss aversion etc. with economic theory; “whereas standard economics is premised on a rational choice model and assumes that individuals make decisions optimally, behavioral economics not only acknowledges that behavior is often suboptimal, but also identifies decision errors and judgmental biases that contribute to such departures from optimality” (6).

The behavior economic field attempts to account for economic irrationality by incorporating psychological factors. Because many people tend to be impulsive, favoring smaller short-term benefits over larger, but more future oriented benefits, the classical economic model fails to accurately predict behavior. People in fact, do not always act in their long-term interests. John et al. (6) write that behavioral economics, “is emerging as a key discipline in modifying self-destructive behaviors, such as those leading to obesity.”

John et al. (6) created a study in which a cohort was motivated to lose weight by means of a deposit contract in which participants deposited “between $0.00 and $3.00 per day of their own funds to a deposit contract. During the month, participants accumulated rewards … equal to his deposit, plus a 1:1 match from the researchers … Participants were aware, however, that they would only receive accumulated awards if they weighed at or below their weight loss goal at the end of the month weigh-in. Thus, these participants could earn $84 net ($168 gross) per month (i.e., by making the maximum $3.00 daily deposit, and … attaining their daily weight loss goal (1 lb per week).” In comparison to control subjects who only met with a dietician, intervention subjects in this study lost over 8 times as much weight (average incentive with loss = 8.70 pounds, mean control = 1.17 pounds).

In this study, John et al. (6) demonstrated that interventions that emphasize small economic rewards, but especially economic loss-aversion can be an important facet in combating obesity. The researchers also demonstrated that even small rewards act as a strong incentive if they occur immediately, demonstrating the tendency to be motivated by a short-term time preference.

## Discounting The Future

According to Rasmussen et al. (7), a formula for delay discounting considering an indifference point at which hyperbolic (short-time preference) discounting occurs may be a useful tool when considering monetary spending which can also be applied to eating behavior. Rasmussen et al. (7) discuss an experiment in which “researchers pose a series of hypothetical choices to participants in which they choose between a relatively small monetary outcome (e.g., $10) available immediately and a larger delayed monetary outcome (e.g., $100).” The researchers gradually then manipulate the “smaller, sooner” reward until reaching a point where the subject “switches from choosing the larger, delayed amount to choosing the smaller, sooner amount” (7). This value or pattern of switching can be described using Mazur's hyperbolic discounting equation (8):

$$\begin{array}{l} V=A/1+\;\text{k}\text{D} \end{array}$$

V is the subjective value of the delayed reward, A is the numerical amount of the delayed reward, D is the time delay, and k is “a free parameter that quantifies the rate of decay of the reward value as delay increases, or the relative degree of discounting” (7).

Rasmussen et al. (7) report that in general, “the value of the outcome is equal to the amount but loses value with delay… higher k values refer to greater sensitivity to delay, or higher impulsivity.” Rasmussen et al. (7) believe that understanding discounting may “prove useful in the development of treatments across a wide array of problems in particular, obesity.”

## More Than Short Term Time Preference: Genetics, Epigenetics, and Environment

It is true that genetic factors influence obesity. Genetics can be of paramount importance as is seen in Prader–Willi, Beckwith–Wiedemann syndrome and other genetic syndromes that lead to obesity. There are also endocrine disorders that result in a small number of pediatric obesity cases.

Over sixty common genetic markers have now been identified as predisposing factors for an increased susceptibility to obesity with thirty-two of the most common genetic variants responsible for ~ <1.5% of the overall inter-individual variation in BMI (9). However, genetic markers in general seem to play a small role (<2%) in the development of obesity.

The field of Epigenetics explores the “phenomena and mechanisms that cause chromosome-bound, heritable changes to gene expression that are not dependent on changes to DNA sequence” (10). Epigenetics is dependent on environmental influences. It is likely that our changing environment may be inducing epigenetic changes that are contributing to higher levels of obesity in the population. “Secular trends in obesity in children, adolescents and adults have shown an increase in obesity with urbanization, clearly indicating the role of the environment” (11).

Nearly all obesity is related to environmental factors that facilitate excess calorie intake (4). As researchers develop a deeper understanding of obesity, epigenetics may ultimately resolve the nature vs. nurture debate. By reconciling these two perspectives, epigenetics may offer new solutions for environmental changes which may decrease the prevalence of obesity in the population.

## Health and Social Costs

Beyond the obvious correlations of obesity with hypertension, hyperlipidemia, type 2 diabetes mellitus, depression and social isolation, obesity is also associated with functional and anatomical brain changes. When compared to those with a lower BMI, obese adults demonstrate frontal lobe, anterior cingulate gyrus, hippocampal, and thalamic brain atrophy (12). Obesity is correlated with poorer cognitive performance in executive functions, especially in impulse control (13).

Obesity increases overall mortality. A study by Mokdad et al. (14) determined that, in the United States, 15% of deaths were attributable to excess weight. Rome (3) notes that “…prevention is paramount because (the) morbidly obese individuals…remain at risk for a shortened lifespan if they do not achieve a significant weight loss.” Obesity in middle age may shorten lifespan by up to seven years (15).

Childhood obesity is particularly dangerous as it sets the pattern for a lifetime. Children who become obese are more likely to remain obese as adults. Adolescents who become obese “…have a 90% chance that their obesity will persist into adulthood” (3). These children are more likely to eat than to spend time with friends (16). They miss or have decreased involvement in important life activities including decreased opportunities for dating, marriage, and reproduction.

## Advertising and Marketing an Unhealthy Diet

Food companies in the US spend billions of dollars annually advertising food products. In 2006, marketing companies spent $1.6 billion advertising food specifically to children (17). In 2004, the average child in the USA viewed 15 food advertisements daily (17). By 2007, American families spent “42% of every food dollar on food prepared by others, up from 25% in 1970.” (18).

Children typically develop emotional connections between themselves and brands that they see advertised (e.g., McDonald's Happy Meal, Joe Camel, or the Marlboro Man) (19). When children are exposed to attractive food triggers, they are stimulated to desire the advertised food and increase its intake (20). Children do not have the sophistication, even with parental guidance, to fully understand the real consequences of consuming what is advertised by the fast-food industries. Children also do not understand obesity's future costs (21).

A tremendous amount of research has been conducted to explore dietary patterns that seem to be the most important factor in the pathogenesis of obesity (22). In particular, “…sugar-sweetened beverages, have received considerable attention largely because added sugar consumption has been rising concomitantly with prevalent obesity” (23). Regulation of sugary beverages provide an example of the possible role of policy interventions in combating the obesity epidemic. Fernandez and Raine (24) report that “sugar sweetened beverage (SSB) taxation is a viable anti-obesity policy. However, researchers and public health practitioners need to be vigilant of industry tactics to curtail SSB lowering efforts.”

## Success in the Past: The Tobacco Model

Since the 1964 release of the Surgeon General's report describing the dangers of tobacco use, smoking prevalence has been cut in half in the United States (25). Media campaigns and anti-smoking laws have been successful in shifting the public's view of smoking as a benign, even attractive habit to a largely stigmatized and undesirable one. In 2009, the Children's Health Insurance Program Reauthorization Act increased cigarette taxes by $0.62 to a total federal tax of $1.01 per pack. Decreased tobacco use prevalence rates afterwards proved “…that increasing the cost of cigarettes is one of the most powerful interventions we can make to prevent smoking and reduce prevalence” (25). Increased taxation was part of a dual pronged approach. The Tobacco Control Act, the second prong in the assault on tobacco use, assessed fees to tobacco manufacturers for sustained public media campaigns targeting youth tobacco prevention and cessation (25).

## Beginning Interventions

In 2014, the Mexican government implemented two policy regulations designed to combat and reverse its obesity incidence rate: taxes on high-calorie foods and drinks; and the restriction of television advertising for high-calorie food and soft drinks between 14:30 and 19:30 on weekdays and between 07:30 and 19:30 on weekends (26). “Overall, 40% of commercials for soft drinks, confectionery and chocolates (will) disappear from TV, in favor of products which “meet nutritional standards,” per the Mexican health ministry.” (26) Norway, the United Kingdom and Quebec have also banned fast food advertising (26).

Although no successful legislation interventions have passed in the United States, there have been attempts to limit the sale of large soft drinks in New York City (27); to increase taxation on fast food, and to eliminate soda sales in schools (4). Some researchers advocate limiting production and importation of sugary beverages along with increased taxation on fast foods; and advocate for fast food restaurant zoning restrictions (4).

## Conclusion: Pediatric Obesity Economic Policy

Hruby and Hu (4) write, “That barely one in three people in the USA today are normal weight portends, quite simply, an astounding and frightening future.” If obesity trends could be reversed, “significant reductions in public health and healthcare expenditures could occur” (28). The most important intervention is the prevention of pediatric obesity itself.

The public health community has demonstrated that public smoking bans, bans on advertising cigarettes to children, and increased taxation on tobacco was successful in decreasing tobacco use in the United States. The same increased taxation and bans on advertising (without outright fast-food bans) could work when applied to the restaurant industry.

While multiple interventions are needed, policies that eliminate problematic environmental triggers (advertising) would likely show an immediate benefit because they combat impulsive/compulsive use and impose a minimal inconvenience (21). If we were to eliminate the marketing, as has been done in Mexico, Quebec, The United Kingdom and elsewhere, and if taxation on fast food were increased—by following the cigarette model—we would have a means to combat the obesity epidemic.

## Author Contributions

NA and BI together conceptualized, collected data, created the formal analysis, methodology, administered the project, and wrote the original and all subsequent drafts.

## Conflict of Interest

The authors declare that the research was conducted in the absence of any commercial or financial relationships that could be construed as a potential conflict of interest.
